# A Robust Composite Proton Exchange Membrane of Sulfonated Poly (Fluorenyl Ether Ketone) with an Electrospun Polyimide Mat for Direct Methanol Fuel Cells Application

**DOI:** 10.3390/polym13040523

**Published:** 2021-02-10

**Authors:** Geng Cheng, Zhen Li, Shan Ren, Dongmei Han, Min Xiao, Shuanjin Wang, Yuezhong Meng

**Affiliations:** 1 The Key Laboratory of Low-carbon Chemistry & Energy Conservation of Guangdong Province/State Key Laboratory of Optoelectronic Materials and Technologies, School of Materials Science and Engineering, Sun Yat-sen University, Guangzhou 510275, China; chengg25@mail2.sysu.edu.cn (G.C.); lizh63@mail2.sysu.edu.cn (Z.L.); stsrs@mail.sysu.edu.cn (S.R.); handongm@mail.sysu.edu.cn (D.H.); stsxm@mail.sysu.edu.cn (M.X.); 2 School of Chemical Engineering and Technology, Sun Yat-sen University, Zhuhai 519082, China

**Keywords:** electrospun mat, proton exchange membrane, direct methanol fuel cell, composite membrane

## Abstract

As a key component of direct methanol fuel cells, proton exchange membranes with suitable thickness and robust mechanical properties have attracted increasing attention. On the one hand, a thinner membrane gives a lower internal resistance, which contributes highly to the overall electrochemical performance of the cell, on the other hand, strong mechanical strength is required for the application of proton exchange membranes. In this work, a sulfonated poly (fluorenyl ether ketone) (SPFEK)-impregnated polyimide nanofiber mat composite membrane (PI@SPFEK) was fabricated. The new composite membrane with a thickness of about 55 μm exhibited a tensile strength of 35.1 MPa in a hydrated state, which is about 65.8% higher than that of the pristine SPFEK membrane. The antioxidant stability test in Fenton’s reagent shows that the reinforced membrane affords better oxidation stability than does the pristine SPFEK membrane. Furthermore, the morphology, proton conductivity, methanol permeability, and fuel cell performance were carefully evaluated and discussed.

## 1. Introduction

Direct methanol fuel cells (DMFCs) are a type of proton exchange membrane fuel cell (PEMFC) and have potential applications in portable electronic devices. They have many advantages over H_2_-O_2_ fuel cells, such as easy storage, easy transportation, utilization of liquid methanol, high energy conversion efficiency at low temperatures, and so on [1,2,3,4]. Proton exchange membranes are of vital importance for PEMFCs by acting both as the electrolytes for proton transport and as separators for isolating the anodes and cathodes and preventing fuel penetration [5,6,7]. Because of the importance shown above, researchers around the world have paid considerable attention to proton exchange membranes (PEMs) in recent years. Among the countless PEMs developed so far, commercially available perfluorinated sulfonic acid membranes (PFSAs, typically Nafion^®^) employed in DMFCs exhibited high proton conductivity, excellent chemical and thermal properties, and acceptable mechanical strength. However, high production cost and methanol permeability of Nafion^®^ hindered its widespread application [8,9,10,11]. So, many researchers turned to developing alternatives to Nafion^®^. Many fluorine-containing [12,13] and non-fluorinated polymers have been developed.

Among the various candidates, non-fluorinated polymers, especially sulfonated aromatic-based polymers, have received considerable attention as alternative PEMs for DMFC application [14,15,16,17,18]. One aromatic-based PEM, sulfonated poly (fluorenyl ether ketone) (SPFEK), was developed by our laboratory previously and has a low production cost (130 USD/cm^2^), acceptable chemical and thermal stability, and excellent methanol barrier ability. However, there are still some drawbacks limiting its application for PEMs. As is well known, due to the lack of a hydrophilic–hydrophobic phase separation domain, sulfonated aromatic-based polymers exhibit generally lower proton conductivity than Nafion^®^. A common way to improve proton conductivity is to attach more sulfonic acid groups onto the polymer chain, but the high degree of sulfonation results in a deterioration of mechanical strength and an increase in methanol permeability because of the excessive membrane swelling and consequent increased electro-osmotic drag [19,20]. Another effective method is to control the thickness of the membrane. According to the work of B. Mullai Sudaroli and co-workers [21], the thickness of the membrane has a significant influence on the polarization curve of DMFCs under real conditions. Thinner membranes achieve lower internal resistance but higher methanol permeation, so an optimal thickness of membrane is beneficial for improving the overall performance of the cell. For SPFEK, the severe deterioration in mechanical properties makes the preparation of thinner membranes difficult.

In more recent years, the electrospinning technique, which can be used for fabricating polymer nanofiber, has been widely employed in the energy field [22,23,24]. The flexible, porous, and robust polymer nanofiber mat prepared by the electrospinning technique is a wonderful substrate for the reinforcement of polymer electrolyte membranes [25,26,27]. A. Manthiram and co-workers [28] fabricated Nafion^®^-impregnated polyvinylidene fluoride (PVDF) nanofiber mat composite membranes where electrospun PVDF membrane was referred as EPM. Composite membranes with the optimal content of Nafion^®^ (EPM/Nafion: 0.4 g) show better cell performance than that of pure Nafion^®^ membranes. Gong and co-workers [29] used polydopamine (PDA)-modified PVDF nanofiber mats as the matrix to fabricate SPEEK/PDA@PVDF composite membranes that exhibited superior DMFC performance (104 mW cm^−1^) compared to that of Nafion 115 due to its better selectivity. Xie and co-workers [30] prepared chitosan filled PVDF/PWA (phosphotungstic acid, PWA) composite membranes by impregnating chitosan into PWA-coated PVDF. Although the composite membrane exhibits a proton conductivity as high as 2.30 × 10^−2^ S cm^−1^, it shows a power density of 85.0 mW cm^−1^, which is worse than that of Nafion^®^ 211.

Although PVDF nano-felt has been utilized in fuel cells to some extent, the modification of the PVDF matrix is a basic prerequisite for preparing composite proton exchange membranes. Two critical obstacles have restricted the extensive application of PVDF in PEMs. On the one hand, electrospun PVDF nanofiber mats are known to exhibit strong hydrophobic behavior, which is not conducive to the effective wetting and impregnation of hydrophilic aromatic-based polymers. Modification of PVDF nanofiber mats is usually needed to improve the hydrophilicity of PVDF [31,32]. On the other hand, PVDF dissolves in strong polar aprotic solvent easily, like many aromatic-based PEM polymers, which complicates the impregnation process. The allocating of mixed solvent is necessary before the impregnation process. Compared with PVDF nanofiber mats, polyimide (PI) nanofiber mats show distinct advantages. The hydrophilicity of PI nanofiber mats makes their impregnation with hydrophilic polymers easier. Besides, the insolubility of PI nanofiber mats simplifies the impregnation process.

In this work, we present the fabrication and characterization of SPFEK-impregnated electrospun polyimide (PI) nanofiber mat composite membranes with optimal thickness and stronger mechanical strength. The preparing process is described in detail. The microstructure, mechanical properties, proton conductivity, methanol permeability, and DMFC single cell performance of the as-prepared membranes were carefully evaluated and discussed.

## 2. Experiment

### 2.1. Materials and Chemicals

Polyimide (PI) electrospun nanofiber mats were kindly supplied by Jiangxi Advanced Nanofiber S&T Co., Ltd. (Jiangxi, Nanchang, China). Basic information of the PI nanofiber mats is given in Table S1. Sulfonated poly (fluorenyl ether ketone) (SPFEK, degree of sulfonation was 60%) was synthesized by the method reported previously [33]. The synthesized process is also described in Scheme S1 of the Supplementary Material. *N*, *N*-dimethylacetamide (DMAc) (A.R.) was purchased from Aladdin Inc. (Montrose, CA, USA). All chemicals were used as received without further treatment.

### 2.2. Fabrication of PI@SPFEK Composite Membrane

**Figure 1:**
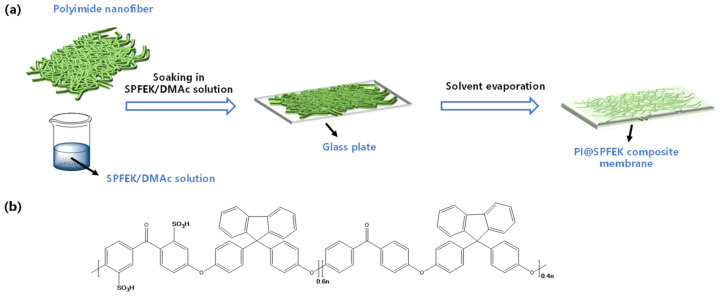
(**a**) Schematic illustration of the fabrication process of the PI@SPFEK membrane; (**b**) Chemical structure of SPFEK. PI@SPFEK is a sulfonated poly (fluorenyl ether ketone) (SPFEK)-impregnated polyimide nanofiber mat composite membrane.

Firstly, SPFEK was dissolved in DMAc to form a homogeneous impregnation solution, which had a solid content of 15–30 wt%. Next, the polyimide (PI) electrospun nanofiber mat was immersed into the solution followed by ultrasound application for 20 min, evacuation in a vacuum drier for 1 h, and ultrasound application for another 20 min so as to achieve the full discharge of bubbles. Then, the solution-impregnated PI electrospun nanofiber mat was transferred to a clean glass plate for solvent evaporation at 60 °C for about 24 h. Finally, the composite membrane, named PI@SPFEK, was peeled from the glass plate by immersing it in deionized-water (DI-water) for several minutes, followed by acidification by 0.5 M H_2_SO_4_ for 24 h at 80 °C. All membranes that were prepared (Figure 1) were washed with DI-water until neutral and stored in DI-water for further evaluation. The thickness of the membrane was controlled by adjusting the solid content of the impregnation solution.

### 2.3. Characterizations of the PI@SPFEK Composite Membrane

#### 2.3.1. Morphology and Structure Characterization

The surface and cross-section morphology of the PI@SPFEK composite membrane and pure SPFEK membrane were examined by a field emission scanning electron microscope (FE-SEM, HITACHI S4800, Hitachi Ltd., Tokyo, Japan). However, due to the introduction of the PI nanofiber mat, it was rather difficult to fracture the composite membrane with liquid nitrogen. So, scissors were used for specimen preparation, and the surface and cross-sectional images of whose cut specimens were observed.

#### 2.3.2. Water Uptake and Swelling Ratio

Firstly, all membranes were dried at 80 °C under vacuum for 24 h, and their weights, lengths, and widths were all measured. Then, all membranes were immersed into DI-water and related parameters were collected under different temperatures (room temperature, 40 °C, 60 °C, 80 °C) after 24 h of balance. The water uptake and swelling ratio were calculated by the following equations:

$$\mathrm{Water}\;\mathrm{uptake}\left(\%\right)=\frac{m_{w}-m_{d}}{m_{d}}\times 100\%$$

$$\mathrm{Area}\;\mathrm{swelling}\left(\%\right)=\frac{L_{w}\times W_{w}-L_{d}\times W_{d}}{L_{d}\times W_{d}}\times 100\%$$

where *m_d_* and *m_w_* refer to the membranes weights before and after being immersed in DI water. The swelling ratio is defined as the percentage increment of membrane area after water absorption. *L_w_* and *L_d_* are the lengths and widths of the hydrated membranes, and *W_w_* and *W_d_* are the lengths and widths of the dried membranes. Three membranes were measured and averaged.

#### 2.3.3. Mechanical Property

Tensile strength was determined under ambient temperature as soon as possible, to avoid the influence of temperature and humidity, by a universal mechanical testing machine (New SANS, Shenzhen, China) at a speed of 5 mm/min [34,35]. The stretching direction was the mechanical direction (MD), and the size of the samples was 1 × 6 cm^2^ under a hydrated state (immersed in DI-water for 24 h before test). Three membranes were characterized and a typical result was presented.

#### 2.3.4. Thermal and Oxidative Stability

The thermal stability was determined by a thermogravimetric analyzer (TGA, Pekin Elmer SII, Waltham, MA, USA) under nitrogen atmosphere with a heating rate of 10 °C/min from room temperature to 800 °C. Fenton’s reagent (3% H_2_O_2_ aqueous solution, 4 ppm FeSO_4_) was used to examine the oxidative stability of the membranes by recording the collapsed time, when the membrane was completely broken into pieces at 80 °C.

#### 2.3.5. Proton Conductivity Test

An electrochemical station (Autolab PGSTAT204, Metrohm, Switzerland) was used to conduct the in-plane proton conductivity test of the composite membrane. The membrane was clamped between two electrodes of a custom-made fixture (Figure S2). Then, the fixture was placed in a constant temperature and humidity chamber (Dongguan Perfect Instrument Co., Ltd. Guangdong, Dongguan, China) for temperature and humidity control. The proton conductivity was calculated by the following equation:

$$\sigma =\frac{L}{R\times A}$$

where *σ* (S/cm) is the proton conductivity. *L* (2 cm in this work) refers to the distance between the two electrodes. *R* (Ω) is the ohmic resistance measured by an electrochemical impedance spectroscope (EIS) over the frequency range from 10 Hz to 1 MHz. The mathematical derivation of how *R* (Ω) was estimated according to Nyquist plots is given in the Supplementary Material (Part 1). *A* (cm^2^) is the cross-sectional area of the membrane. The testing was applied to three membranes, and a representative result was shown.

#### 2.3.6. Methanol Permeability

Two methods were used to investigate the methanol permeability of the composite membrane, and a typical result was presented.

1. Traditional diffusion method (cyclic voltammetry, CV): The membrane was clamped between custom-made equipment with two-compartment diffusion cells in which equivalent amounts of methanol/sulfuric acid solution (5 M/0.5 M) and sulfuric acid solution (0.5 M) were added, respectively. A cyclic voltammetry method was used to determine the concentration of methanol in the diffusion cell. The methanol permeability can be calculated according to the following equation:
  $$P=\frac{l}{A}\times \frac{V}{C_{0}}\times \frac{\Delta C}{\Delta t}$$
  where *P* is the methanol permeability (cm^2^ s^−1^). *l* and *A* are the thickness (cm) and area (cm^2^) available for permeation, respectively. *V* and *C*_0_ are the volume (cm^3^) and initial concentration (mol L^−1^) of the methanol solution, respectively. Δ*C*/Δ*t* is the slope of the methanol concentration varying with time in the water compartment [36,37,38]. The set-up is shown in Figure S3. An example for the calculation of the *P* value is also given in the Supplementary Material (Part 2).
2. Linear sweep voltammetry method (LSV method): During the measurement, nitrogen gas was introduced to the cathode with a flow rate of 100 mL min^−1^. A positive voltage range from 0 to 1.0 V was applied using an electrochemical workstation (Autolab PGSTAT302N, Metrohm, Switzerland) while a methanol solution (2M) was injected into the anode side at a flow rate of 1 mL min^−1^. The methanol crossover was determined by measuring the limited current density produced by the complete electro-oxidation of methanol permeation at the cathode side.

#### 2.3.7. Membrane Electrode Assembly (MEA) Preparation

A commercially available gas diffusion electrode (Shanghai Hesen Electric Co., Ltd., Shanghai, China) with a catalyst loading of 4 mg cm^−2^ Pt-Ru/C for the anode and 2 mg cm^−2^ Pt/C for the cathode was used for the membrane electrode assembly (MEA) preparation. The sandwich structure of the MEA was prepared by hot pressing at 140 °C for 2 min with a loading of 1 MPa. To reduce the interface resistance effectively, the surface of the membrane was brush-coated with a Nafion 212/DMAc solution, which had a solid content of 2%, using a Chinese writing brush before hot pressing. The effective area of the membrane was 6.25 cm^−2^.

#### 2.3.8. Single Cell Performance Evaluation

All membranes were evaluated by an Arbin fuel cell testing system (Arbin Instrument Inc., College Station, TX, USA) where the anode was supplied with a 2 M methanol aqueous solution at a flow rate of 2 sccm while the cathode was fed with humidified pure O_2_ at a flow rate of 500 sccm. The single cell was activated by a constant-voltage activation method at 0.4 V for 2 h and tested at 80 °C. Three specimens were evaluated, and a reproducible result was given.

## 3. Results and Discussion

### 3.1. Preparation and Morphology Characterization of PI@SPFEK

**Figure 2:**
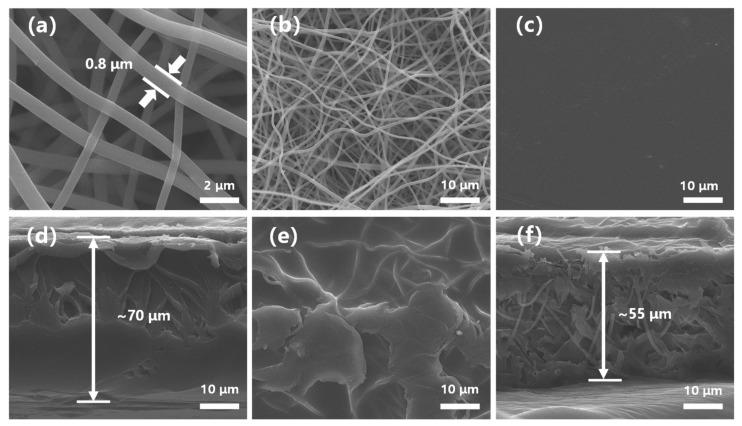
Scanning electron microscope (SEM) images of (**a**,**b**) polyimide (PI) nanofiber, (**c**,**d**) SPFEK, and (**e**,**f**) PI@SPFEK for top surfaces and cross-sections, respectively.

Figure 2 represents the top surface and cross-sections of the polyimide nanofiber mat, SPFEK, and PI@SPFEK composite membranes, respectively. From Figure 2a,b, the morphology of the PI nanofiber was clearly exhibited and the fibers were interwoven. Figure S4 is a cross-sectional image of the polyimide nanofiber at a different magnification. The PI nanofiber mat we used had a thickness about 55 μm. The SPFEK membrane had a smooth surface (Figure 2c) and dense internal structure (Figure 2d). For the PI@SPFEK composite membrane, the PI nanofibers can be clearly observed in the internal structure, which indicates that the framework of the nanofiber mat was not destroyed during the solution-impregnation procedure. The composite membrane (Figure 2f) had a similar thickness (ca. 55 μm) to that of the original PI nanofiber mats. At a higher magnification (Figure S5), we can see that the PI nanofibers were surrounded by polymers. The voids in Figure S5 are characteristic of technical problems. The stress in the scissoring process for SEM sampling resulted in the formation of voids. It should be noted that the top surface of the composite membrane (Figure 2e) is much rougher compared with that of the pure SPFEK membrane. This phenomenon is closely associated with the preparation process, especially the solid content of the SPFEK/DMAc solution for impregnation. The 15% solid content of the SPFEK/DMAc solution was chosen for impregnation because the higher solid content may lead to the encapsulation of nanofiber mats, like that seen in amber, due to the increasing thickness and lower solid content, which results in incomplete filling of the SPFEK into the voids of the nanofiber mat.

### 3.2. Thermal and Oxidative Stability

**Figure 3:**
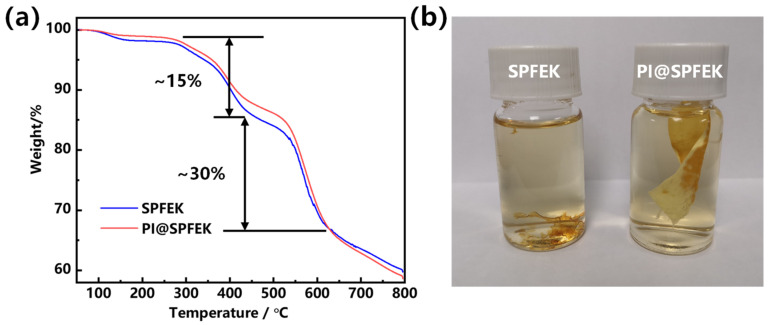
(**a**) Thermogravimetric (TG) analysis curves of SPFEK and PI@SPFEK composite membranes; (**b**) Photograph of SPFEK and PI@SPFEK composite membranes after 1 h of treatment with Fenton’s reagent (3% H_2_O_2_, 4 ppm FeSO_4_) at 80 °C.

Thermogravimetric (TG) analysis was used for the thermal stability evaluation of SPFEK and PI@SPFEK composite membranes. According to Figure 3a, a two-step degradation profile was observed for these two membranes. The weight loss at about 280 °C can be attributed to the loss of the sulfonic acid group, and a higher degradation temperature at about 500 °C contributed to the breakdown of the backbone of the polymer. SPFEK and PI@SPFEK composite membranes exhibit a similar weight-loss curve due to the low weight content of PI in the composite membrane (only 7 wt% as measured).

Proton exchange membranes are easily attacked by free radicals generated under real running conditions of DMFCs. A proton exchange membrane with high oxidative stability is desperately needed. Fenton’s reagent was used for the treatment of these membranes at 80 °C to evaluate their antioxidative stability. According to our experiment, SPFEK was easily collapsed into pieces after about 1 h of treatment (Figure 3b). For PI@SPFEK, it remained intact without any loss of weight after 1 h, which may be attributable to the confinement effect of the PI nanofiber mat. However, due to the chemical component of SPFEK in PI@SPFEK, the composite membrane must eventually change, and a break-down of SPFEK is inevitable. After about 3 h, SPFEK was completely dissolved out of the composite membrane with only the framework of the nanofiber mat remaining, indicating the strong resistance to oxidation of the PI nanofiber and the extensive potential for its use in fuel cells.

### 3.3. Water Uptake and Swelling Ratio of Membranes

**Figure 4:**
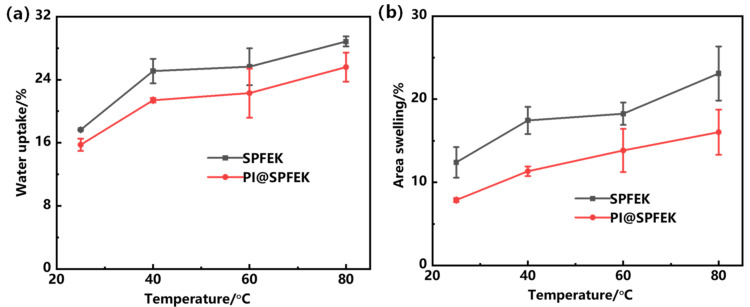
(**a**)Water uptake and (**b**) area swelling rate of SPFEK and PI@SPFEK at different temperatures.

The water uptake is closely related to the proton conductivity, methanol permeability, and mechanical properties of proton exchange membranes. Due to the similar transportation mechanism of protons and methanol molecules, water uptake of a membrane is a double-edged sword. More water molecules absorbed are beneficial for the formation of dense hydrogen bond networks and hydrated hydrogen ions, which can accelerate the transmission of protons and improve proton conductivity [39]. However, excessive absorption of water will not only expedite methanol permeation but also deteriorate the mechanical properties. Figure 4 shows the water uptake and area swelling of SPFEK and PI@SPFEK composite membranes at different temperatures. Obviously, water uptake of the two membranes increased with increasing temperature. This is because the polymers we used, except the polyimide nanofiber mat, have hydrophilic side chains but hydrophobic backbone. So, the hydrophilic–hydrophobic phase separation domains can form in membranes. With increasing temperature, the polymer chains moved more violently and caused an extended phase separation domain, which accommodated more water molecules. So, the water uptake increased with temperature. Moreover, SPFEK showed higher water uptake at all temperatures compared with that of the PI@SPFEK composite membrane. Swelling ratio is another important parameter used for the evaluation of dimensional stability of membranes, which is of great significance for the practical operation of DMFCs. Compared with the SPFEK membrane, the PI@SPFEK composite membrane showed a lower area of swelling at each temperature. The better dimensional stability of the PI@SPFEK composite membrane is attributed to the limitation of the three-dimensional framework of the PI nanofiber mat.

### 3.4. Mechanical Performance 

**Figure 5:**
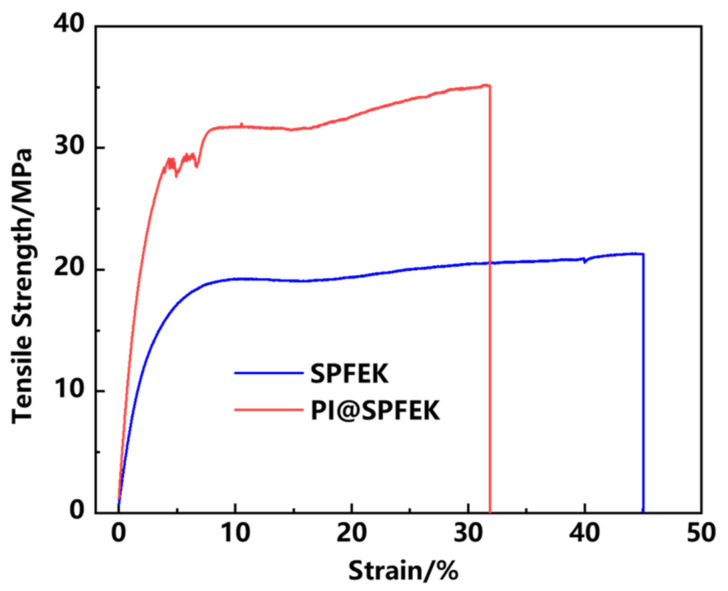
Tensile stress–strain curves of SPFEK and PI@SPFEK in a hydrated state.

The mechanical properties of the proton exchange membrane seriously affect the life span of a membrane under real application conditions and then affect the structural stability of the MEA. In a hydrated state, the PI@SPFEK composite membrane showed a tensile strength up to 35.1 MPa, which is far higher than that of the pure SPFEK membrane with a tensile strength of 21.3 MPa (Figure 5). The remarkable improvement of tensile strength gives an outstanding rigidity to the composite membrane because of the introduction of the PI nanofiber mat. The results provide an effective method for the improvement of mechanical properties.

### 3.5. Proton Conductivity and Methanol Permeability

**Figure 6:**
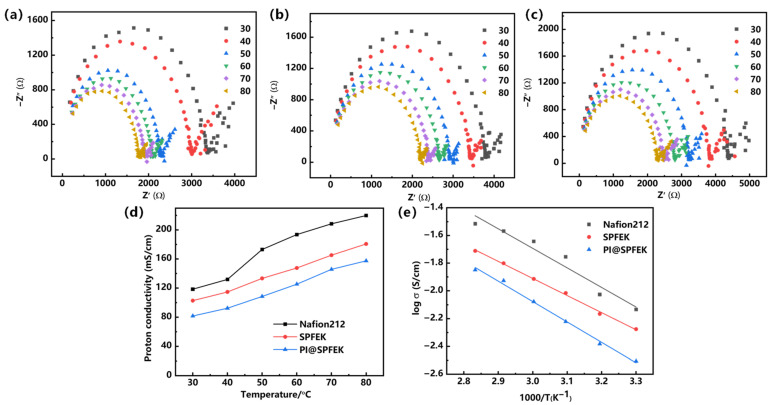
Nyquist plots of (**a**) Nafion 212, (**b**) SPFEK, and (**c**) PI@SPFEK. (**d**) Temperature dependence of proton conductivity of membranes and (**e**) Arrhenius plots of membranes. The cross-sectional area of Nafion 212, SPFEK, and PI@SPFEK composite membrane used for the proton conductivity tests were about 5 × 10^−^^3^, 5 × 10^−3^, and 7.0 × 10^−3^ cm^2^, respectively.

Proton conductivity is a decisive parameter for PEMs and is closely related to the applicability of PEMs. For easier comparison, the commercial Nafion 212 was evaluated under the same condition as those for the SPFEK and PI@SPFEK membranes. Figure 6a–c illustrates the Nyquist plots of the three membranes at different temperatures, which all represent two feature regions with a semicircle in the high-frequency zone and a nearly linear section in the low-frequency zone. Figure 6d,e shows the temperature dependence of proton conductivity for three different membranes under 100% RH (relative humidity) and related Arrhenius plots. It can be seen that both SPFEK and PI@SPFEK exhibited lower proton conductivity than that of Nafion 212, but they all satisfy the proton conductivity needed for real applications. PI@SPFEK showed the lowest proton conductivity but is comparable with that of SPFEK. PI@SPFEK gives an activation energy of 12.2 kJ/mol, which is slightly higher than the value of 10.2 kJ/mol for SPFEK. This is reasonable sincethe introduction of proton non-conductible PI can hinder the proton conduction and lead to a decline of proton conductivity and an increase in activation energy.

**Figure 7:**
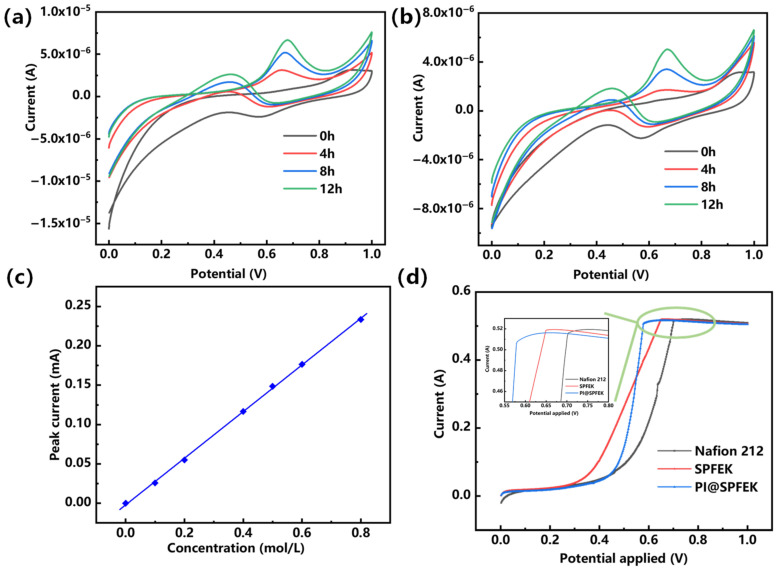
Cyclic voltammograms curves of (**a**) SPFEK and (**b**) PI@SPFEK membranes at room temperature over different diffusion times; (**c**) Calibration curve of the methanol solution; (**d**) The linear sweep voltammetry (LSV) curves of membranes for methanol crossover testing.

The crossover of methanol from the anode to the cathode can reduce the fuel conversion efficiency and result in low energy density and power density. Two electrochemical methods were used for the evaluation of methanol crossover. Figure 7a,b gives the CV (cyclic voltammetry) curves of the SPFEK and PI@SPFEK membranes by a traditional diffusion method over different diffusion times at room temperature. The methanol oxidation peak current is positively correlated with methanol content in solution. We can clearly see that peak current increased with time, indicating the unavoidable penetration of methanol throughout the membrane. Comparing the peak currents of the SPFEK and PI@SPFEK membranes, we can see that the PI@SPFEK membranes have a lower peak current under the same penetration time, which means they have a better methanol barrier ability. The methanol permeability of SPFEK and PI@SPFEK is 2.36 × 10^−8^ cm^2^ s^−1^ and 1.94 × 10^−8^ cm^2^ s^−^^1^, respectively. These values were calculated according to the calibration curve given in Figure 7c and are only 60% and 48%, respectively, of the methanol permeability of Nafion 212 (3.98 × 10^−8^ cm^2^ s^−1^). The LSV method was also applied to qualitatively assess the methanol permeability of those membranes. It can be clearly seen in the inset of Figure 7d, that PI@SPFEK shows the lowest peak current compared to that of Nafion 212 and SPFEK. It is well known that methanol permeability has a similar change trend to that of proton conductivity as methanol and protons share the same transportation mechanism. The hypothesis is well verified from above two methods.

### 3.6. Single Cell Performance

**Figure 8:**
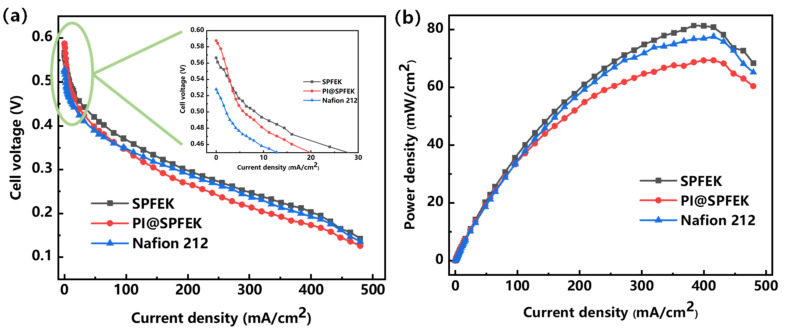
Polarization (**a**) and power density (**b**) curves of SPFEK and PI@SPFEK composite membrane at 80 °C with a 2 M methanol solution.

For the purpose of evaluating the overall performances of SPFEK and PI@SPFEK under a real application condition, the MEAs from our membranes were fabricated and several single fuel cells were further assembled. The performance of these MEAs and others made from Nafion 212 were tested at 80 °C. Figure 8 gives the polarization and power density curves of SPFEK, PI@SPFEK, and Nafion 212. The PI@SPFEK composite membrane has an open circuit voltage of 0.59 V, which is the highest compared with those of SPFEK and Nafion 212, indicating the lowest methanol permeability is for PI@SPFEK (Inset of Figure 8a). SPFEK and Nafion 212 membranes show a similar polarization curve. For the PI@SPFEK membrane, the introduction of proton non-conductive PI nanofiber mats reduces the proton conductivity and exhibits a comparatively higher polarization than that of SPFEK and Nafion 212. As for power density (Figure 8b), SPFEK gives the best performance due to it having lower methanol permeability and acceptable proton conductivity. The results of the single cell performances of SPFEK, PI@SPFEK, and Nafion 212 are in good accordance with the discussion in Section 3.5. As a robust proton exchange membrane, the PI@SPFEK composite membrane shows acceptable and even higher power density when compared with that reported for other PEMs for DMFC applications [40,41].

## 4. Conclusions

In this work, a PI@SPFEK composite membrane was successfully prepared via impregnating a PI nanofiber mat in a synthesized SPFEK solution. The as-made PI@SPFEK composite membrane shows better anti-oxidant stability, lower water uptake, and stronger dimensional stability compared with those of the original SPFEK membrane. The PI@SPFEK membranes also exhibit improved mechanical strength and superior methanol resistance ability. MEAs derived from the as-synthesized membrane were fabricated, and several single fuel cells were assembled. The performance of these single cells based on the PI@SPFEK composite membrane had the highest open circuit voltage of 0.59 V because of their low methanol permeability. They also showed high power density when compared with that reported for other PEMs for DMFC applications.

## Data Availability

The authors confirm that the data supporting the findings of this study is available within the article.

## Supplementary Materials

The following are available online at https://www.mdpi.com/2073-4360/13/4/523/s1, Table S1: Basic information of the PI nanofiber mat, Scheme S1: Synthesis procedure of SPFEK, Figure S1: Optical photograph of the PI@SPFEK composite membrane, Figure S2: (a) Front view and (b) top view of the custom-made fixture, Figure S3: Set-up used for the methanol permeability measurement, Figure S4: Cross-sectional image of the PI nanofiber mat, Figure S5: A higher magnification image of the PI@SPFEK composite membrane cross-section, Figure S6. Remaining CV data of (a) SPFEK and (b) PI@SPFEK membranes, Part 1: How R (Ω) was estimated according to Nyquist plots is given below, Figure S7: Schematic of R value obtained, Part 2: The way to calculate the P value of the methanol permeability. Taking PI@SPFEK composite as an example. References [36,37,38] are cited in the supplementary materials.
