# Supplementary material for: A Robust Composite Proton Exchange Membrane of Sulfonated Poly (Fluorenyl Ether Ketone) with an Electrospun Polyimide Mat for Direct Methanol Fuel Cells Application
Source: Polymers (Basel). 2021 Feb 10;13(4):523. doi: 10.3390/polym13040523 (PMC7916468; doi:10.3390/polym13040523)
Supplement: Supplementary file 1 [file polymers-13-00523-s001.pdf]

polymers

MDPI

Supplementary Material: A robust composite proton exchange membrane of SPFEK with electrospun polyimide mat for DMFC application

Geng Cheng, Zhen Li, Dongmei Han, Min Xiao, Shuanjin Wang, Yuezhong Meng

Table S1. Basic information of PI nanofiber mat.

**Thickness (μm)**	**Density (g/cm³)**	**Tensile strength (MD, MPa)**	**Tensile strength (TD, MPa)**
55±5	0.188	11.24	9.80
Intensity of acupuncture (N)	Breathability (s)	Elongation at break (MD, %)	Elongation at break (MD, %)
0.52	0.157	29.53	28.19
Voltage resistance (V)	Porosity (%)	Pore size distribution (μm)	The main aperture (μm)
150	86.58	3.305-4.406	3.769

The schematic for synthesis of SPFEK was presented below. Briefly, to a 50 mL three-necked round-bottomed flask equipped with a condenser, a Dean-stark trap and a magnetic stirrer, BPF (0.7008 g, 2 mmol), SDFBP (0.5068 g, 1.2 mmol), DFBP (0.1769 g, 0.8 mmol), anhydrous potassium carbonate (0.35g, 2.5 mmol), DMSO (5 mL) and toluene (6 mL) were added. Under the protection of nitrogen, the mixture was refluxed at 150°C for 5 h. After that, water was azeotroped off with toluene, followed by heated at 170°C for 20 h. Finally, the mixture was cooled to ambient temperature and precipitated in mix solution of methanol and water. The precipitates were filtered and washed thoroughly with DI-water. The fibrous polymer was dried at 120°C under vacuum for 24 h and collected for further application.

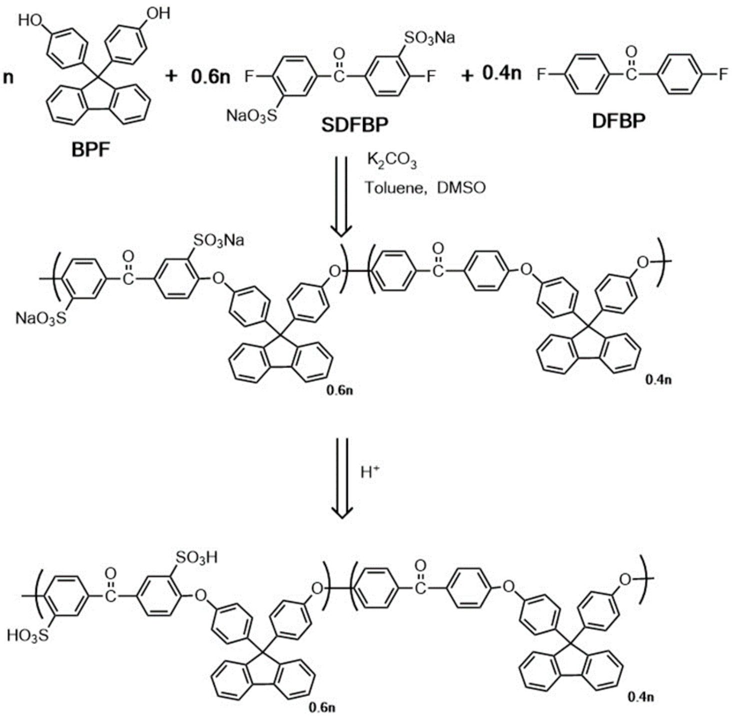

Scheme S1. Synthesis procedure of SPFEK.

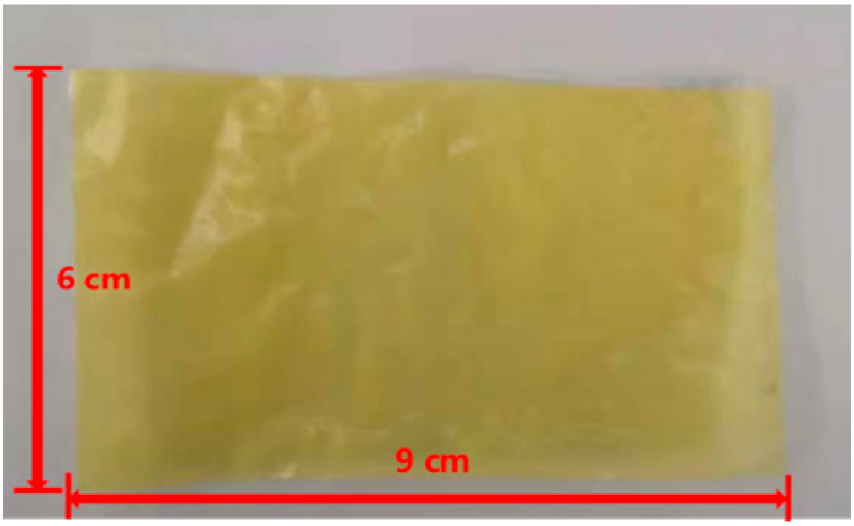

Figure S1. Optical photograph of PI@SPFEK composite membrane.

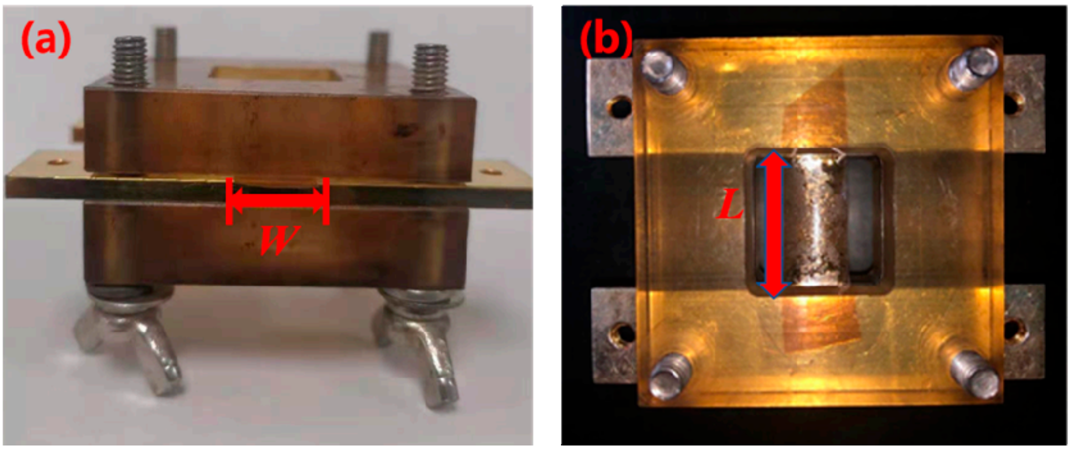

Figure S2. (**a**) Front view and (**b**) top view of the custom-made fixture.

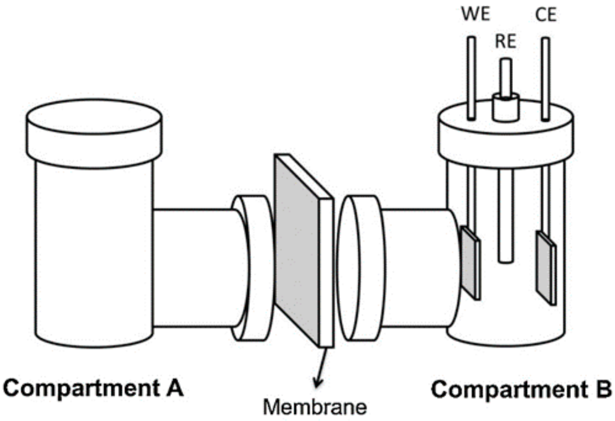

Figure S3. Set-up used for methanol permeability measurement.

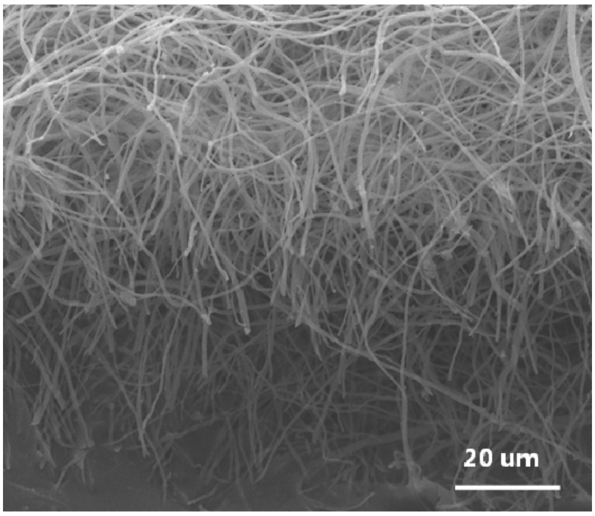

Figure S4. Cross-sectional image of PI nanofiber mat.

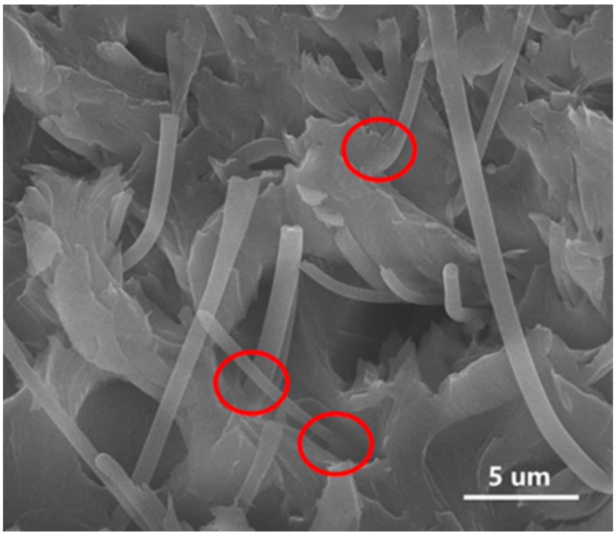

Figure S5. The higher magnification image of PI@SPFEK composite membrane cross-section.

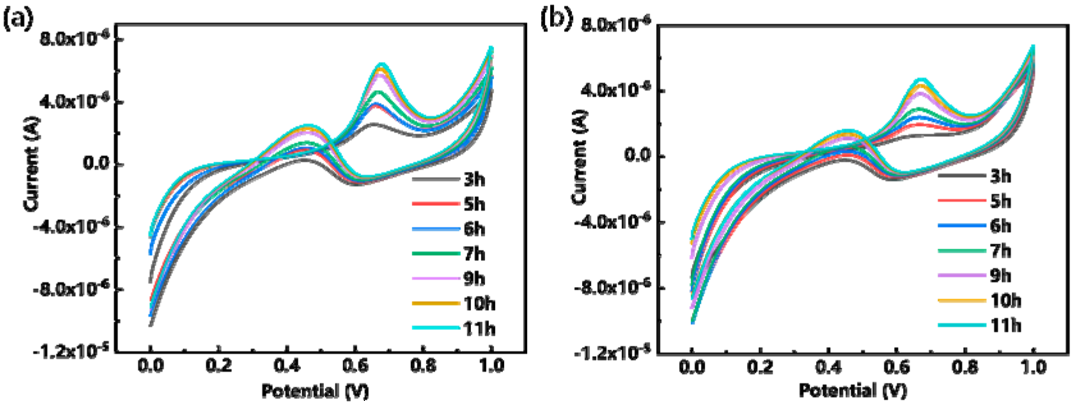

Figure S6. Remaining CV data of (**a**) SPFEK and (**b**) PI@SPFEK membranes.

Part 1: How *R* (Ω) was estimated according to Nyquist plots was given below.

R is the core parameter for proton conductivity calculation. Firstly, the Nyquist plots was obtained *via* an AC impedance method. The *R* value was acquired by fitting the Nyquist plots. The diameter of the semicircle obtained by fitting is regarded as the *R* value.

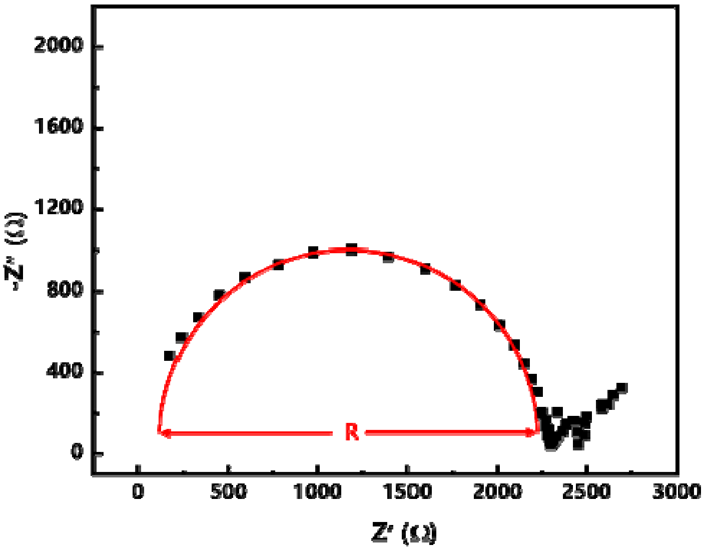

Figure S7. Schematic of R value obtained.

Why the diameter of the semicircle obtained by fitting can be regarded as *R* value. That's because the equivalent circuit we used for the evaluation of the measured impedance spectra is an RC parallel circuit. For an RC parallel circuit, the diameter of the semicircle is considered as the R value. The mathematical derivation is presented below.

The complex impedances of the resistor and capacitor under AC conditions present like this respectively:

$$\dot{Z}_R = R \tag{1}$$

$$\dot{Z}_C = -\frac{1}{j\omega C} \tag{2}$$

j is imaginary unit. The reciprocal of the total complex impedances of the RC parallel circuit is equal to the sum of the reciprocal of the complex impedances of the two parallel elements.

$$\frac{1}{\dot{Z}} = \frac{1}{\dot{Z}_R} + \frac{1}{\dot{Z}_C} = \frac{1}{R} + j\omega C = \frac{1+jR\omega C}{R} \tag{3}$$

$$\dot{Z} = \frac{R}{1+jR\omega C} = \frac{R}{1+(\omega RC)^2} - \frac{j\omega R^2 C}{1+(\omega RC)^2} = Z' + jZ'' \tag{4}$$

So, the real and imaginary parts of impedance are given below.

$$Z' = \frac{R}{1+(\omega RC)^2} \tag{5}$$

$$Z'' = \frac{\omega R^2 C}{1+(\omega RC)^2} \tag{6}$$

The tangent of the phase angle can be acquired.

$$tan\theta = \frac{Z''}{Z'} = -\omega RC \tag{7}$$

Substituting equation (7) into equation (5) or (6), and eliminating the angular frequency ω, equation (8) can be obtained.

$$(Z' - \frac{R}{2})^2 + Z''^2 = (\frac{R}{2})^2 \quad (8)$$

This is the equation of a circle in the complex plane with a centre at (*R*/2, 0) and a radius of *R*/2. That's the reason why the diameter of the semicircle obtained by fitting can be regarded as *R* value.

Part 2: The way to calculate *P* value of methanol permeability. Taking PI@SPFEK composite as an example.

Figure S3 gives the set-up used for methanol permeability measurement, and the ***P*** value is calculated according to the following equation.

$$P = \frac{l}{A} \times \frac{V}{C_0} \times \frac{\Delta C}{\Delta t} \quad (1)$$

l is the thickness of the composite membrane, which is about 49.3 μm here. ***A*** is the effective area of membrane for methanol, which is 1.767 cm^2 here. ***V*** is the initial volume of solution in compartment B, it's 70 mL constantly for all measurement. C_0 is the initial concentration of methanol solution in compartment A, which is also a 5 M methanol solution constantly for all measurement. The data acquirement of $\Delta C/\Delta t$ is a little sophisticated. Firstly, a series of standard methanol solution were prepared. CV scanning was applied to these solutions, and the peak currents can be obtained according to theses CV curves. Then, the calibration curves can be obtained (Figure 7c in the revised manuscript). The equation is as follows:

$$y = 0.3x - 0.002 \quad (2)$$

In the initial phase, this equation is nearly can be obtained.

$$\frac{\Delta C}{\Delta t} = \frac{C-0}{t-0} \quad (3)$$

So, the peak current at 5h of PI@SPFEK membrane is used for further calculation. According to equation (2), the C of PI@SPFEK membrane after 5h of penetration is 8.96×10^{-4} mol L^{-1}. So, the value $\Delta C/\Delta t$ is 4.98×10^{-8} mol L^{-1} s^{-1}.

P value can be acquired according to l, *A*, *V*, C_0 and $\Delta C/\Delta t$, which is 1.94×10^{-8} cm^2 s^{-1}.

References

1. Xue, S.; Yin, G. Methanol permeability in sulfonated poly(etheretherketone) membranes: A comparison with Nafion membranes. *Eur. Polym. J.* **2006**, *42*, 776–785, doi:10.1016/j.eurpolymj.2005.10.008
2. Zaidi, S.M.J. Comparative Study of Electrochemical Methods for Determination of Methanol Permeation Through Proton-Exchange Membranes. *Arab. J. Sci. Eng.* **2011**, *36*, 689–701, doi:10.1007/s13369-011-0085-1
3. Almeida, T.P.; Miyazaki, C.M.; Paganin, V.A.; Ferreira, M.; Saeki, M.J.; Perez, J.; Riul, A.; Jr, A.R. PEDOT:PSS self-assembled films to methanol crossover reduction in Nafion ® membranes. *Appl. Surf. Sci.* **2014**, *323*, 7–12, doi:10.1016/j.apsusc.2014.08.056
